# Retraction notice to Gadolinium-Free Cardiac MR Stress T1-Mapping to Distinguish Epicardial From Microvascular Coronary Disease: J Am Coll Cardiol 71 (2018) 957-968

**DOI:** 10.1016/j.jacc.2020.08.038

**Published:** 2020-10-20

**Authors:** Alexander Liu, Rohan S. Wijesurendra, Joanna M. Liu, Andreas Greiser, Michael Jerosch-Herold, John C. Forfar, Keith M. Channon, Stefan K. Piechnik, Stefan Neubauer, Rajesh K. Kharbanda, Vanessa M. Ferreira

**Affiliations:** Oxford Centre for Clinical Magnetic Resonance Research, Division of Cardiovascular Medicine, Radcliffe Department of Medicine, University of Oxford, Oxford, United Kingdom; Siemens Healthcare GmbH, Erlangen, Germany; Brigham and Women’s Hospital, Radiology, Cardiovascular Imaging, Boston, Massachusetts; Oxford Heart Centre, John Radcliffe Hospital, Oxford, United Kingdom; Division of Cardiovascular Medicine, Radcliffe Department of Medicine, University of Oxford, Oxford, United Kingdom

This article has been retracted: please see Elsevier Policy on Article Withdrawal (https://www.elsevier.com/about/our-business/policies/article-withdrawl).

The *JACC* Journals Ethics Board has voted to retract this paper, relying on the findings of misconduct after an investigation by the University of Oxford (outlined below).The decision to retract the paper follows the conclusion of an investigation under the University of Oxford’s (“the University’s”) Code of Practice and Procedure on Academic Integrity in Research (“the Code”). The Registrar of the University convened a Panel under the Code. The Panel considered a number of issues, including in relation to this paper. The Panel concluded that the first author, Dr Alexander Liu, was responsible for misconduct in research. The Panel’s findings with regards to misconduct were limited to the actions of the first author. No other co-author was found to be involved in the misconduct. It is understood that the first author disagrees with the Panel’s findings. The first author has raised a complaint with the Office of the Independent Adjudicator for Higher Education (OIA) (The OIA reviews complaints from students about their higher education provider).

In relation to this paper, the Panel’s findings included that:

• certain data had been fabricated by the first author amending the actual study data so that the paper and the central illustration would show a compelling case that T1 mapping could distinguish between epicardial obstructive coronary artery disease and coronary microvascular dysfunction;
• the number of control subjects, their age, and the statistical test to calculate the significance of a difference between the patients in this paper were incorrect; and
• Figure 2 had been fabricated.

The Panel’s view was that this paper would likely need to be retracted from the literature as it had major irregularities and its conclusions were unsafe.

The following co-authors agree that a retraction is appropriate: Vanessa Ferreira, Rajesh Kharbanda, Stefan Neubauer, Stefan Piechnik, Keith Channon, John C. Forfar, Michael Jerosch-Herold, Andreas Greiser, Joanna Liu and Rohan Wijesurendra.

